# Status and related factors of burnout among palliative nurses in China: a cross-sectional study

**DOI:** 10.1186/s12912-022-01083-x

**Published:** 2022-11-15

**Authors:** Yalin Zhang, Jianjun Jiang, Chuanmei Zhu, Chunhua Liu, Chang Guan, Xiaolin Hu

**Affiliations:** 1 West China School of Nursing, Sichuan University/Department of Nursing, West China Hospital, Sichuan University, Chengdu, China; 2 Department of Palliative Medicine, West China School of Public Health and West China Fourth Hospital, Sichuan University, Chengdu, China; 3 Outpatient Department, West China Hospital, Sichuan University, Chengdu, China; 4 Department of Biotherapy, Cancer Center, West China Hospital, Sichuan University, Chengdu, China

**Keywords:** Burnout, Cross-sectional study, Nurses, Palliative care, Risk factors

## Abstract

**Background:**

Burnout occurs frequently in nurses and seems more common in nurses providing palliative care. However, to our knowledge, there is a lack of understanding regarding the factors influencing burnout among palliative nurses in China.

**Methods:**

A cross-sectional design was conducted. A total of 331 palliative nurses from 25 hospitals participated in this study. Anonymous data were collected through a self-designed social-demographic questionnaire, the Nursing Burnout Scale, the Perceived Social Support Scale, the General Self-Efficacy Scale, the Connor-Davidson Resilience Scale, and the Simplified Coping Style Questionnaire. Independent sample *t* tests, one-way ANOVA, Pearson correlations, and multiple linear regressions were performed to identify the related factors of the three dimensions of burnout.

**Results:**

In the results of multiple linear regression, resilience, health condition, coping style, and pessimistic personality were common related factors; in addition, end-of-life care training, social support, and income satisfaction were statistically significantly associated with burnout. These factors could explain 38.6%, 27.8%, and 34.5% of the total variance in emotional exhaustion, depersonalization, and reduced personal accomplishment, respectively.

**Conclusions:**

The findings of this study help policy makers and nurse managers better understand burnout among palliative nurses in China. The results highlighted the importance of implementing culture-oriented training programs, providing perceived organizational support, and building a reasonable salary system to decrease burnout among palliative nurses, increase the quality of nursing and promote the development of Chinese palliative care.

## Introduction

Burnout is a condition and a psychological syndrome defined as emotional exhaustion, depersonalization, and reduced personal accomplishment related to work stressors [1]. A wealth of research has confirmed that burnout occurs frequently in health care professionals, including doctors, nurses and social workers, among whom, nurses are especially susceptible to burnout [2]. From previous studies, 54%-68.3% of nurses are deem to burnout [3, 4], leading to psychosomatic symptoms such as decreased energy, fatigue, anxiety, depression, and negative evaluation of personal accomplishments [5, 6]. In addition, nurse burnout can independently predict patient safety, adverse events [7], and increased hospital infections [8].

Palliative care is a care approach provided to patients (and families) with life-threatening illness that aims to identify, assess, and treat pain and other problems, including physical, mental and spiritual issues, to improve their quality of life [9]. Palliative care work is considered to be innately challenging. Nurses working in palliative units provide services to patients in the terminal period and their families and are more frequently exposed to grief, suffering and death than other medical staff are [10]. As a result, palliative nurses seem to face more emotional distress and psychological problems than general nurses do, and many studies have demonstrated that nurses working in palliative units have higher levels of burnout [11, 12].

Burnout in palliative nurses may result in a negative attitude towards palliative care, increased turnover rates, decreased nursing care quality, and reduced hospitalization satisfaction among patients [3, 13], which may indirectly hinder the development of palliative care. According to the WHO, an estimated 40 million people need palliative care each year (WHO, 2020), and China may account for a relatively large part of this figure, given its population of more than 1.4 billion [14]. However, palliative care is developing relatively slowly in China, and hospice care services covers only 10% of the population [15]. In addition to the policy and legal situations, the cultural context in China may account for it. Confucian culture holds the perspective that “life and death are preordained” and attributed to fate [16], resulting in a lack of attention to palliative care. In addition, talking about death is somewhat taboo in traditional Chinese culture [16], and palliative nurses may have difficulty confiding in their family and friends about emotional problems caused by their end-of-life care work. These situations suggest that palliative nurses in China may tend to experience job burnout to a certain degree, and their negative attitudes could be an important factor in the poor quality of Chinese palliative care [17]. Therefore, paying close attention to Chinese palliative nurses’ burnout is urgent.

In previous studies, some individual features, such as age [18], personality [19] and inner resilience [20], and some social-occupational factors, including years of experience [21], social support [22] and job training [12], have been demonstrated to be factors of nurse burnout. However, few studies have been conducted in palliative contexts, and to our knowledge, no studies have focused on the influencing factors of burnout in Chinese palliative nurses. Considering the relevant large difference in Chinese conditions and culture compared with other countries, it is necessary to fill this gap. Thus, we conducted this cross-sectional study to 1) investigate burnout status among Chinese palliative nurses, and 2) to identify the factors associated with burnout.

## Methods

### Design, setting, and participants

A cross-sectional design with convenience sampling method was performed in this study. From May 2021 to September 2021, we investigated a total of 340 palliative nurses from 25 hospitals or healthcare institutions in 8 cities, and 331 questionnaires were recycled; the response rate was 97.35%. Two independent researchers used an author-designed Excel spreadsheet for data entry, and this was verified by a third researcher. This study was part of our cross-sectional study about palliative nurses in China, other details about participants’ selection process please see our previous work [23].

### Procedure

After acquiring permissions to conduct this study from the nurse managers of each selected hospitals or healthcare institutions, two well-trained researchers assigned the questionnaires to palliative nurses by face. Subjective information was collected in the form of paper questionnaires, and objective information was obtained through the personnel management system with the help of human resource managers. Staff meetings were regularly conducted under the lead of nursing managers in each participating palliative unit to promote the data collection process and to ensure the response rate. The signed informed consent forms and the completed questionnaires were required to return together and were accessible only to researchers of this study.

### Sample size

The sample size was analysed using G*power 3.1 software, and the linear multiple regression algorithm was selected. There were 21 variables, including 12 sociodemographic characteristics and nine scale-associated dimensions, in this study. With 95% confidence intervals and 0.8 power, the minimum sample size in our study was 160. Considering a possible 15% wastage rate, a total of 188 participants were needed.

### Measures

#### Social-demographic characteristics

We used a self-designed questionnaire to collect information on the participants’ social-demographic characteristics, including their gender, age, educational level, marital status, religion, health condition, chronic disease, family support, professional title, years of work experience, monthly income, income satisfaction, and participation in end-of-life care training.

#### Burnout and pessimistic personality

The burnout and pessimistic personality subscales of the Chinese version of Nursing Burnout Scale (NBS) was used to assess burnout and pessimistic personality of participants in this study [24]. This scale uses a 4-point Likert scale with response options ranging from 1 referring to “strongly disagree” to 4 referring to “strongly agree”. The 12-item burnout subscale comprises of 3 dimensions of nurse burnout (emotional exhaustion, depersonalization, reduced personal accomplishment), with each dimension comprising 4 items. The total scores ranged from 12–48 points, and a higher score reflected a higher level of burnout. In this study, the Cronbach’s α the three dimensions were 0.91, 0.85, and 0.88, respectively. The 12-item personality subscale has the total scores ranged from 12–48 points, with higher scores indicating that the participants were more likely to have pessimistic personality. The Cronbach’s α of the scale in this study was 0.76.

#### Social support 

The Perceived Social Support Scale (PSSS) was used to measure nurses’ social support [25]. The scale comprises 12 self-report items, providing a subjective assessment of the individual’s social support from his or her family, friends and other connections. A Likert-type scoring (1–2-3–4-5–6-7) is applied, and the total scores range from 12 to 84 points, with higher scores indicating more social support perceived by participants. The Cronbach’s α for the PSSS was 0.96 in this study.

#### Self-efficacy

We used the General Self-Efficacy Scale (GSES) to assess self-efficacy among palliative nurses. The Chinese version [26] of the GSES is a 10-item single-dimension scale, and each item is scored on a 4-point Likert-type scale (1 = not true at all, 2 = true, 3 = mostly true, 4 = totally true), with total scores ranging from 10–40 points. A total score less than 20 points is classified as a low level of self-efficacy, 20–30 points is a medium level of self-efficacy, and more than 30 points is identified as a high level of self-efficacy. The GSES has been proven to have acceptable internal reliability, and the Cronbach’s α of the scale in this study was 0.90.

#### Resilience

The resilience level of nurses was measured by the Connor-Davidson Resilience Scale (CD-RISC). This scale is a 25-item Guttman scale assessing 3 domains, including tenacity, strength and optimism, with response options ranging from 0–4 (0 = not true at all, 1 = rarely true, 2 = sometimes true, 3 = often true, 4 = always true). The total score ranges from 0–100, and a higher score indicates a higher level of resilience. The Chinese version of the CD-RISC has been proven to have acceptable reliability (Cronbach’s α = 0.75) and could be a screening tool for resilience [27]. In this study, Cronbach’s α was 0.95.

#### Coping style

Coping style was evaluated using the Simplified Coping Style Questionnaire (SCSQ). The SCSQ was developed by Xie in 1998 [28] and consists of 20 items focusing on coping cognitive and behavioural patterns over 2 domains: the positive coping dimension (items 1–12) and the negative coping dimension (items 13–20). The items are rated using a 4-point Likert scale ranging from 0 for “not used” to 3 for “used a great deal”. The average scores of the two dimensions were calculated, and the higher the average score of the dimension was, the greater the tendency to adopt the coping style. In this study, the Cronbach’s α of the SCSQ and its 2 dimensions were 0.86, 0.87 (positive coping dimension) and 0.85 (negative coping dimension).

### Statistical analysis

We used SPSS 26.0 for data analysis. Skewness and kurtosis were used for normality tests. The variables were determined to have a normal distribution when the skewness value ≤ 2 or the kurtosis value ≤ 4 [29], and we found that our data (scores for emotional exhaustion, depersonalization, reduced personal accomplishment, pessimistic personality, social support, self-efficacy and coping style) were normally distributed. The independent-sample t test (dichotomous variables) and one-way ANOVA (polytomous variables) were conducted for univariate analysis. Correlations among dimensions of subscales were analysed using Pearson correlation analysis. Finally, multiple linear regression analysis was carried out to separately test the related factors of emotional exhaustion, depersonalization and reduced personal accomplishment dimensions. We chose stepwise regression analysis to minimize the multicollinearity effect (“the alpha to enter” and “the alpha to remove” were set as 0.05 and 0.1, respectively). The multicollinearity in this regression model was checked using tolerance, and the variance inflation factor (VIF), with tolerance < 0.1 or VIF > 10, indicated multicollinearity. The result was considered statistically significant when the two-tailed *p* value was less than 0.05.

## Results

### Characteristics of participants

As shown in Table 1. The mean age and the mean years of work experience of the 319 nurses were 30.88 ± 7.22 years and 5.88 ± 5.9 years, respectively. Most of the participants were female (98.43%), and the majority were married (62.70%). The most common educational level was undergraduate or above (53.61%), and the most common professional title was junior nurse (73.89%). Only one-third of participants (31.39%) reported being satisfied with their income.

**Table 1:** Patients’ characteristics

Variables	N/M	%/SD
Age	30.88	7.22
Length of working service	5.88	5.9
Gender		
Male	5	1.57%
Female	314	98.43%
Educational level		
Junior college	148	46.39%
Undergraduate and above	171	53.61%
Marital status		
Unmarried	119	37.30%
Married	200	62.70%
Religion		
Yes	17	5.33
No	302	94.67%
Health condition		
Very bad	4	1.25%
Bad	10	3.13%
General	139	43.57%
Good	124	38.87%
Very good	42	13.17%
Chronic disease		
Yes	33	10.34%
No	286	89.66%
Professional title		
Junior	236	73.98%
Intermediate and above	83	26.02%
Monthly income		
< 3,000 yuan (US, $500)	49	15.36%
3,000–5,000 yuan (US, $500–$830)	137	42.95%
5,000–7,000 yuan (US, $830–$1,160)	92	28.84%
7,000–9,000 yuan (US, $1,160–$1,500)	35	10.97%
≥ 9,000 yuan (US, $1,500)	6	1.88%
Income satisfaction		
Very bad	9	2.82%
Bad	53	16.61%
General	157	49.22%
Good	86	26.96%
Very good	14	4.39%
Participate in end-of-life care training		
Yes	258	80.88%
No	61	19.12%
Burnout (total)	23.4	7.68
Emotional exhaustion	8.66	3.04
Depersonalization	7.46	2.58
Reduced personal accomplishment	7.29	2.83
Pessimistic personality	30.89	3.85
Social support	61.26	11.73
Self-efficacy	23.36	5.30
Resilience	57.12	14.60
Coping style		
Positive dimension	2.00	0.59
Negative dimension	1.32	0.65

The mean scores of burnout total and emotional exhaustion, depersonalization, and reduced personal accomplishment dimensions were 23.4 ± 7.68, 8.66 ± 3.04, 7.46 ± 2.58, and 7.29 ± 2.83, respectively. The mean score of pessimistic personality was 30.89 ± 3.85. In addition, the mean scores of social support, self-efficacy and resilience were 61.26 ± 11.73, 23.36 ± 5.30, and 57.12 ± 14.60, respectively, suggesting that palliative nurses in this study generally had high levels of social support and moderate levels of self-efficacy and resilience [25–27]. For the coping style, the mean scores for the positive dimension and negative dimension were 2.00 ± 0.59 and 1.32 ± 0.65, respectively, which means that most palliative nurses tend to adopt a positive coping style [28].

### Bivariate analysis between nurse burnout and sociodemographic variables

Bivariate analysis results suggested that educational level, health condition, family support, income satisfaction and end-of-life care training were statistically significantly associated with all three dimensions (*p* < 0.05) (Table 2).

**Table 2:** Bivariate analyses ^a^

	**Emotional exhaustion**			**Depersonalization**			**Reduced personal accomplishment**		
	***M* ± *SD***	***t***	***F***	***M* ± *SD***	***t***	***F***	***M* ± *SD***	***t***	***F***
Educational level		2.343*			2.225*			2.455*	
Junior college	9.08 ± 2.95			7.80 ± 2.63			7.70 ± 2.74		
Undergraduate and above	8.29 ± 3.08			7.16 ± 2.50			6.93 ± 2.86		
Health condition			7.695^**^			6.098^**^			8.043^**^
Very bad	15.00 ± 1.16			12.50 ± 3.11			14.00 ± 3.37		
Bad	10.10 ± 3.84			8.50 ± 2.59			7.80 ± 3.39		
General	9.06 ± 3.00			7.74 ± 2.51			7.60 ± 2.77		
Good	8.00 ± 2.84			7.06 ± 2.35			6.73 ± 2.61		
Very good	8.31 ± 2.75			7.00 ± 2.84			7.12 ± 2.56		
Income satisfaction			8.458^***^			3.218^***^			6.401^***^
Very bad	11.67 ± 4.44			8.22 ± 3.99			9.44 ± 4.80		
Bad	9.72 ± 3.02			7.79 ± 2.44			8.26 ± 2.94		
General	8.83 ± 2.83			7.71 ± 2.50			7.34 ± 2.65		
Good	7.72 ± 2.81			7.02 ± 2.49			6.74 ± 2.55		
Very good	6.50 ± 2.79			5.64 ± 2.68			5.00 ± 2.29		
Participate in end-of-life care training		-2.359^*^			-2.047^*^			-2.149^*^	
Yes	8.46 ± 3.06			7.32 ± 2.55			7.12 ± 2.79		
No	9.48 ± 2.84			8.07 ± 2.65			7.98 ± 2.89		

^a^ Only results with significant correlations have been listed

^*^ p < .05; ^**^ *p* < .01; ^***^ *p* < .001

### Correlations of burnout among palliative nurses

The results of Pearson correlation analyses are presented in Table 3. Among the three subscales, the correlations were statistically significant (*r* = 0.699, *r* = 0.742, *r* = 0.762, all *p* < 0.01). Among the three dimensions, there were five common influencing factors. Specifically, pessimistic personality and negative coping style were positively correlated with emotional exhaustion, depersonalization, and reduced personal accomplishment (*r* > 0, *p* < 0.01). In addition, social support, resilience and positive coping style were negatively correlated with three dimensions (*r* < 0, *p* < 0.01), while no statistically significant correlations were found among the three dimensions and self-efficacy, age and years of work experience.

**Table 3:** Results of Pearson correlations analysis

Variables	Emotional exhaustion	Depersonalization	Reduced personal accomplishment	Pessimistic personality	Social support	Self-efficacy	Resilience	Positive coping	Negative coping
Emotional exhaustion	1								
Depersonalization	0.699^**^	1							
Reduced personal accomplishment	0.742^**^	0.762^**^	1						
Pessimistic personality	0.245^**^	0.176^**^	0.202^**^	1					
Social support	-0.229^**^	-0.273^**^	-0.274^**^	-0.024	1				
Self-efficacy	-0.116^*^	-0.080	-0.012	0.146^**^	0.125^*^	1			
Resilience	-0.365^**^	-0.301^**^	-0.287^**^	0.007	0.302^**^	0.601^**^	1		
Positive coping	-0.248^**^	-0.236^**^	-0.307^**^	-0.028	0.281^**^	0.298^**^	0.497^**^	1	
Negative coping	0.306^**^	0.272^**^	0.320^**^	0.048	0.016	0.361^**^	0.21^**^	0.182^**^	1

^*^ *p* < .05.; ^**^ *p* < .01

### Multiple linear regression results of nurse burnout

In this study, we included variables with statistically significant correlations in the bivariate analysis and Pearson correlation analysis into multiple linear regression analyses for the three dimensions. In addition, age was included as a common confounding variable [30]. The multiple linear regression results of the relationships among the three dimensions and selected variables in palliative nurses are listed in Table 4.

**Table 4:** Multiple liner regression results of nurse burnout

	**Variables**	***B***	***β***	***t***	***R***^***2***^	**adjusted *R***^***2***^	***F***
**Emotional exhaustion**	Constant	8.163			0.399	0.386	29.539^***^
	Resilience	-0.068	-0.325	-6.171^***^			
	Negative coping	1.743	0.370	8.083^***^			
	Pessimistic personality	0.182	0.230	5.188^***^			
	Income satisfaction	-0.518	-0.143	-3.070^**^			
	Health condition	-0.496	-0.131	-2.824^**^			
	Positive coping	-0.470	-0.105	-2.049^*^			
	Training	0.681	0.088	1.987^*^			
**Depersonalization**	Constant	9.184			0.291	0.278	21.367^***^
	Resilience	-0.043	-0.244	-4.253^***^			
	Negative coping	1.352	0.338	6.881^***^			
	Social support	-0.033	-0.148	-2.906^**^			
	Pessimistic personality	0.111	0.166	3.453^**^			
	Health condition	-0.448	-0.140	-2.839^**^			
	Positive coping	-0.430	-0.113	-2.017^*^			
**Reduced personal accomplishment**	Constant	9.099			0.360	0.345	24.961^***^
	Negative coping	1.682	0.380	8.043^***^			
	Positive coping	-0.923	-0.219	-4.100^***^			
	Resilience	-0.035	-0.179	-3.268^**^			
	Pessimistic personality	0.133	0.179	3.922^***^			
	Income satisfaction	-0.358	-0.105	-2.172*			
	Social support	-0.030	-0.123	-2.509*			
	Health condition	-0.375	-0.105	-2.204^*^			

^*^ *p* < .05.; ^**^ *p* < .01.; ^***^p < .001

The results indicated that resilience, coping style, and pessimistic personality were common related factors of three dimensions (*p* < 0.05), with resilience (*β* = -0.325, *β* = -0.244, *β* = -0.179, all *p* < 0.01) and positive coping (*β* = -0.105, *β* = -0.113, *β* = -0.219, all *p* < 0.01) presented as negative factors, while pessimistic personality (*β* = 0.230, *β* = 0.166, *β* = 0.179, all *p* < 0.01) and negative coping (*β* = 0.370, *β* = 0.338, *β* = 0.380, all *p* < 0.01) were presented as positive factors. Regarding the emotional exhaustion model (*R *^*2*^ = 0.399, adjusted *R *^*2*^ = 0.386, *F* = 29.539, *p* < 0.01), income satisfaction (β = -0.143, *p* = 0.001) and end-of-life care training (no compared yes, *β* = 0.088, *p* = 0.048) were also related factors, and a total of 38.6% of the variance could be explained. In the depersonalization model (*R *^*2*^ = 0.291, adjusted *R *^*2*^ = 0.278, *F* = 21.367, *p* < 0.01), social support also reflected a related factor, and 27.8% of the total variance was explained. In the reduced personal accomplishment model (*R *^*2*^ = 0.360, adjusted *R *^*2*^ = 0.345, *F* = 24.961, *p* < 0.01), income satisfaction and social support were also revealed as related factors, and a total of 34.5% of the variance was explained. There was no multicollinearity found in the three models.

## Discussion

### Burnout status

Findings from correlations between three subscales revealed that palliative nurses who experience emotional exhaustion also experience depersonalization and reduced personal accomplishments, which was consistent with the findings of a previous study [31]. In addition, compared with studies using the same burnout scale, the scores in this study seemed higher than those of Chinese general nurses [32]: 21.65 ± 6.09, *p* < 0.05). In addition to the influence of sample size and the variation across individuals, there may be other reasons for these differences. First, the risk of burnout in medical staff has been demonstrated to be strongly correlated with the frequency of contact with death and suffering [33]. Nurses working in palliative units experience the frequent salvage, hospice care and death of patients, as well as constant grief, helplessness and even despair [34], which places them at a high risk of burnout. In addition, the specific cultural context may make it difficult for palliative nurses to obtain favourable social support. The relatively slow development of Chinese palliative care can also lead to the lopsided coverage and imperfect discipline development in this field [14]. As a result, palliative nurses in China seem to be a more vulnerable group of job burnout than general nurses. Thus, it is urgent to identify burnout among palliative nurses and provide effective strategies to cope with it.

### Common factors of the three dimensions

In this study, coping strategy was strongly associated with emotional exhaustion, depersonalization, and reduced personal accomplishment, which was consistent with the findings of previous studies [35, 36]. Coping is the cognitive and behavioural reactions exhibited to master, tolerate, and reduce stress [37]. Palliative nurses who adopt negative coping strategies such as cognitive avoidance can experience negative outcomes, including psychosomatic symptoms [38] and immune and endocrine system damage [39]. However, positive coping, such as problem solving, positive attitudes, cognitive flexibility, and optimism, can help them be less emotionally exhausted, depersonalized and complaint prone [35, 40], thus reducing their vulnerability to burnout. Therefore, coping strategy training targeted to end-of-life care should be conducted widely with nurses working in palliative units.

Resilience is another strong protective factor that has been found in many other studies as well [20, 21]. Resilience is defined as the ability to cope with adverse circumstances and minimize distress [41]. Nurses with good resilience may regard problems as a normal part of life and adopt ways of thinking that lessen the impact of clinical dilemmas [40]. Moreover, the development of resilience can also facilitate the adoption of positive coping [42], which can reduce burnout synergistically. Thus, effective interventions need to be explored and used to cultivate internal resilience and help palliative nurses survive in high-stress settings.

In addition, personality traits such as neuroticism, negative affectivity, and cooperativeness were demonstrated to have a negative influence on burnout by influencing the way people experience and cope with stressors [43]. Considering this, palliative nurses with a pessimistic personality may be less likely to be happy at work because they tend to interpret their work environment as more negative. They more easily suffer further step distress and social disengagement and are caught in a downwards spiral of burnout [44]. However, as one’s personality is difficult to change or modify, more intention can be focused on other factors when considering practical intervention, and it might be necessary to take personality as an admission qualification of palliative nurses.

Moreover, in this study, the self-rated health condition also revealed strong relations. This function could be explained by the interaction between burnout and health outcomes. According to current review [45], burnout can directly affect health conditions through neuroendocrine mechanisms to deplete the resources necessary for coping, leading to negative health outcomes such as fatigue, depression, and somatization. As for health problems caused by burnout, nurses may not be able to achieve the desired work performance, thus forming a vicious cycle [46]. The impact of burnout on health outcomes found in this study highlighted the importance of identifying health conditions early and adopting preventive interventions in palliative working environments.

### Other factors

Palliative nurses who had accepted end-of-life care training received lower emotional exhaustion scores in this study, which highlights the importance of job training in palliative care. Palliative care work requires strong professional knowledge and skills. However, nearly 20% of the participants in this study reported they have received no end-of-life care training, which might result in incompetent and ineffective care and a lack of emotion, communication, and stress management skills [35]. In addition, under the influence of Confucian culture, people in China rarely talk about death, which might indirectly result in the shortage of palliative medical staff and the slow development of palliative care in China. Therefore, death education should be fully covered for palliative care staff in order to adjust their attitude towards hospice care and death, and it is urgent to extend this out to the public to improve their cognition and understanding of and support for palliative work and thus further promote the development of Chinese palliative care.

In this study, social support and income satisfaction also showed statistically significant correlations with burnout. For nurses, the relationship between healthcare-related occupational stress and burnout can be mediated by perceived social support [47]. In addition, for palliative nurses, support from the work environment, such as perceived organizational support and peer support, seemed especially effective for decreasing their burnout [22]. Thus, it is noted that nurse managers in palliative units should provide support resources for palliative nurses and create a harmonious working atmosphere to help them deal with stress and difficulties.

Past review has confirmed that salary is an important factor for nurses’ job satisfaction [48]. In the US, nurses are paid an average of 98,190 USD/year [49]. However, in China, nurses are paid only 9,500–15,100 USD/year [18], revealing a notable gap in nurse salaries between China and developed countries. In our study, palliative nurses were generally paid 5,600–9,500 USD/year, which was even lower than the Chinese average. The disparity between pay out and income surely contributes to their risk of burnout and intention to leave the field [18]. Thus, a carefully considered and reasonable salary system needs to be established to gradually improve palliative nurses’ job satisfaction.

Some other factors did not manifest significance in this study, including age, educational level, and self-efficacy. It was noted that nurses with older age are vulnerable to burnout due to the reduction of physical and psychological work abilities [50]. However, the majority of nurses in this study were at young age (30.88 ± 7.22), and age did not seem to be the main reason for their burnout. In addition, this study coded educational level as a dichotomous variable (junior college/ undergraduate and above), failing to clarify a more detailed relationship between educational level and burnout, and further studies with larger sample sizes should be conducted to identify such relationships in palliative nurses. Some publications have explained that nurses with higher self-efficacy experienced lower level of burnout [51, 52], which was not detected in this study. This might due to the reason that Chinese palliative nurses take on a relatively overloaded caring tasks, and it is hard for them to overcome work stressors through themselves’ personal abilities [53].

### Implications

Our results suggest that training programs directed at the Chinese cultural context, including death education, coping strategies training, and resilience cultivation, are badly needed to help palliative nurses adjust their cognition and attitudes regarding palliative care, as well as to improve the quality of nursing care and decrease burnout among such nurses; this point is important for nursing managers in formulating an effective training system. Second, palliative nurses with pessimistic personalities and poor health conditions in this study were more likely than other nurses to experience burnout. As such factors were relatively stable and are hard to change [19], it should be noted that the corresponding entry qualifications should be developed before potential palliative nurses are recruited. Third, palliative care policy is relatively limited in China, and the current policy stipulates palliative standards, including structure, human resources, and service environment [14]. However, to address the shortage of palliative nurses through policy solutions and developing localized palliative care in China, policy makers and nurse managers should take particular care to optimize the current policy in light of relevant cultural characteristics and build favourable teamwork, a harmonious atmosphere, and a reasonable salary system in the Chinese context [19, 20].

### Limitations

First, due to the nature of the cross-sectional design, we cannot identify causal relationships among burnout and its influencing factors. Future longitudinal studies are therefore warranted to robustly analyse the correlative relationships among these factors. Second, although we included nearly all palliative nurses from Sichuan Province, the representativeness of the results might be affected due to the lack of samples from other areas in China. As one year has passed since the data was collected, the timeliness of the results may be affected. Third, although information on the possible influencing factors were collected to the best of our knowledge, the results explained less than half of the total variance; thus, more exploratory studies should be carried out in the future.

## Conclusion

This study identified the factors associated with burnout among Chinese palliative nurses. The results revealed that resilience, pessimistic personality, coping strategy and health condition are common factors of three dimensions; in addition, income satisfaction, end-of-life care training, and social support can also affect burnout. In palliative nurse recruitment, personality and health condition can be considered as part of the profession’s entry qualifications. In addition, strategies for reducing burnout include taking culture-oriented training programs (death education, resilience cultivation, and coping skills promotion), providing perceived support resources, and building a reasonable salary system; these can help decrease palliative nurses’ burnout, increase the quality of nursing and promote the development of Chinese palliative care.

## Data Availability

The datasets generated and/or analysed during the current study are not publicly available due to the authors are still working on them but are available from the corresponding author on reasonable request.
